# Dietary Potassium Intake and Risk of Diabetes: A Systematic Review and Meta-Analysis of Prospective Studies

**DOI:** 10.3390/nu14224785

**Published:** 2022-11-12

**Authors:** Lanfranco D’Elia, Maria Masulli, Francesco P. Cappuccio, Aquilino F. Zarrella, Pasquale Strazzullo, Ferruccio Galletti

**Affiliations:** 1 Department of Clinical Medicine and Surgery, “Federico II” University of Naples Medical School, 80131 Naples, Italy; 2 World Health Organization Collaborating Centre for Nutrition, Warwick Medical School, University of Warwick, Coventry CV4 7AL, UK; 3 Department of Medicine, University Hospital Coventry and Warwickshire NHS Trust, Coventry CV2 2DX, UK

**Keywords:** dietary potassium, potassium intake, potassium consumption, diabetes, glucose metabolism, meta-analysis

## Abstract

(1) Background: Dietary potassium intake is positively associated with reduction of cardiovascular risk. Several data are available on the relationship between dietary potassium intake, diabetes risk and glucose metabolism, but with inconsistent results. Therefore, we performed a meta-analysis of the prospective studies that explored the effect of dietary potassium intake on the risk of diabetes to overcome these limitations. (2) Methods: A random-effects dose–response meta-analysis was carried out for prospective studies. A potential non-linear relation was investigated using restricted cubic splines. (3) Results: A total of seven prospective studies met the inclusion criteria. Dose–response analysis detected a non-linear relationship between dietary potassium intake and diabetes risk, with significant inverse association starting from 2900 mg/day by questionnaire and between 2000 and 5000 mg/day by urinary excretion. There was high heterogeneity among studies, but no evidence of publication bias was found. (4) Conclusions: The results of this meta-analysis indicate that habitual dietary potassium consumption is associated with risk of diabetes by a non-linear dose–response relationship. The beneficial threshold found supports the campaigns in favour of an increase in dietary potassium intake to reduce the risk of morbidity and mortality. Further studies should be carried out to explore this topic.

## 1. Introduction

Potassium (K) is one of the major intracellular cations of the human body; indeed around 98% of the body’s K is in intracellular fluid. K plays a key role in several physiological mechanisms, especially in the neuro-endocrine system and in the regulation of blood pressure (BP).

K is one of principal minerals for human nutrition. The main sources of dietary K are vegetables, fruits and dairy products. In foods, K occurs as a mixture of organic and inorganic compounds. Several experimental and clinical studies found an inverse relationship between dietary K intake and BP [1], and a number of investigations showed a favourable effect of a K-rich diet on cardiovascular health, in part independently of effects on BP [2,3,4]. Therefore, these data support the biological plausibility for an influence of dietary K intake on cardiovascular morbidity and mortality.

In this context, different experimental studies also pointed out a favourable effect of K intake on metabolism, in particular on diabetes and glucose metabolism via involvement of the renin-angiotensin-aldosterone system (RAAS) [5], so as contribute to the explanation of the beneficial role of dietary K intake on cardiovascular diseases. On the other hand, epidemiological studies reported contrasting results [6,7,8,9,10,11,12]. In addition, a meta-analysis of prospective observational studies found no significant association between K intake and diabetes risk [13]. However, this meta-analysis assessed the highest versus lowest consumption comparison, included few studies exploring the non-linear association by dose–response analysis, and it did not assess the effect of the risk of bias [14]. Some intervention trials were carried out to investigate the potential effect of K supplementation on glucose metabolism [15,16]. However, also on this occasion the results were not consistent.

Therefore, considering the worldwide prevalence and projected future incidence of diabetes [17], the limitations of the previous meta-analysis above cited, the generally low amount of dietary K intake [18,19,20,21,22], and the increasing body of new evidence on this issue, we decided to perform a new systematic review and meta-analysis of the prospective observational studies. If at all possible, we wanted to explore the relationship between dietary K intake and the risk of diabetes, exploring the shape and strength of the dose–response relationship for these associations.

## 2. Materials and Methods

### 2.1. Data Sources and Search Strategy

This meta-analysis was planned, conducted and reported according to the PRISMA statement [23] (Supplemental Table S1). A systematic search of the available publications was performed using MEDLINE/PubMed, Web of Science, and Scopus, up to August 2022. The search strategy, without restrictions, included the expressions “potassium intake” OR “dietary potassium” OR “potassium supplementation” OR “potassium excretion” AND “glucose” OR “diabetes” OR “insulin sensitivity” OR “insulin resistance” OR “HOMA” OR “insulin” OR “glicated haemoglobin” OR “glycated hemoglobin”, or combinations thereof, either in medical subject headings or in the title/abstract. In addition, a manual search of references from recent reviews and relevant published original studies was performed.

### 2.2. Study Selection and Data Extraction

The study was conducted in accordance with the PRISMA statement [23] by L.D. and A.F.Z. The study protocol was preregistered (CRD42022335517). Inclusion criteria were checked through the titles and abstracts of the studies retrieved in the searches. The full texts of the potentially eligible studies were then evaluated. Discrepant evaluations were resolved in conference with a third reviewer (FG). Data was then extracted from the studies meeting the inclusion and exclusion criteria by L.D. and A.F.Z. in accordance with the PRISMA statement.

### 2.3. Inclusion Criteria

To be included in the meta-analysis, a published study had to meet the following criteria, stratified by study design:

Prospective studies: (a) original articles, (b) studies conducted in the adult population, (c) studies reporting the dietary K intake evaluation at baseline, (d) studies in which the participants have a diagnosis of diabetes which is determined prospectively as outcomes, (e) studies reporting the number of participants exposed and the rate or incidence of diabetes in different categories of K intake, (f) studies in which there is an assessment of risk estimation (hazard ratio—HR, relative risk—RR, odds ratio—OR) for specified K intake categories and (g) studies with a follow-up of at least 2 years (mean or median).

### 2.4. Risk of Bias

The risk of bias of the studies included was assessed according to predefined criteria: the Newcastle–Ottawa Scale for prospective studies [24].

### 2.5. Grading of Evidence

The quality of evidence was evaluated by using the GRADE (grading of recommendations assessment, development and evaluation) methodology [25]. Evidence was graded as high, moderate or low quality. Observational studies were judged as low by default. They were downgraded or upgraded according to prespecified criteria. Criteria to downgrade included study limitations (risk of bias), inconsistency (substantial unexplained heterogeneity), indirectness (factors that limit generalizability), imprecision (95% CI cross a minimally important difference of 5%) and publication bias (significant evidence of small-study effects). Criteria to upgrade certainty of evidence included a large magnitude of effect, a dose–response gradient and attenuation by plausible confounding factors.

### 2.6. Statistical Analysis

The assessment of linear and non-linear association between dietary K intake or urinary K excretion and incidence of diabetes was carried out after adjustment for the greatest number of potential confounders. The possibility of a non-linear relationship was explored by modelling K intake using restricted cubic splines with three knots at fixed percentiles (10%, 50%, and 90%) of K intake distribution. Departure from linearity was assessed by testing the null hypothesis that the coefficient of the second spline was equal to zero. A two-stage dose–response random-effects meta-analysis was performed [26], which takes into account the correlation between the estimates across categories of K intake. The median consumption for each specific category was assigned to each corresponding estimate. If the median or mean consumption was not reported by the authors, the midpoint between the upper and lower boundary was used. If the lowest category was open-ended, its lower boundary was set to zero. If the upper boundary of the highest category was left unspecified, we assumed the category to be of the same amplitude as the preceding one. Statistical heterogeneity across the studies was also explored by Q-test. The pooled analysis combined the results of all studies, but in the case of multiple results [12], only those of the urinary K excretion were included to avoid overlapping, while after stratification by type of dietary assessment (i.e., questionnaire or urinary excretion), the analysis included the results from only that type of assessment.

Next, to explore potential sources of heterogeneity and additional analyses, from the dose–response analysis of a single study, we calculated the risk of diabetes for an increase in 1,000 mg/day in dietary K intake. Funnel plots and formal tests (i.e., Egger’s and Begg’s tests) were performed to explore potential publication bias. In case of significant funnel plot asymmetry, the pooled estimate was recalculated by the “trim and fill” method. In addition, although there was a small number of the included studies in the single meta-analysis, a meta-regression analysis was used to identify possible sources of heterogeneity. To convert urinary output into dietary intake, the urinary excretion of K in mmol/day was first converted to mg/day (1 mmol = 39 mg). Then, the K values were multiplied by 1.3, assuming that around 70% of K ingested is excreted in the urine.

All statistical analyses were performed using the Stata Corp. software (version 11.2; College Station, TX, USA).

## 3. Results

**Figure 1:**
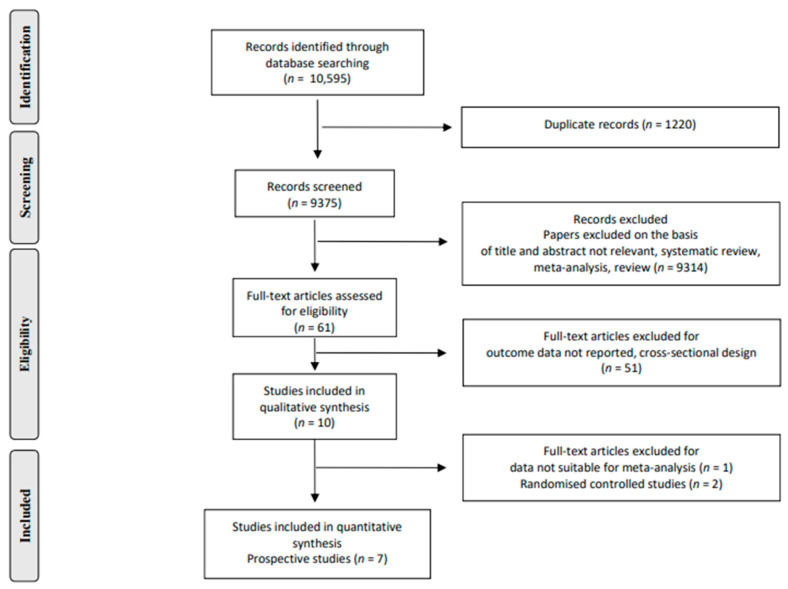
Stepwise procedure for selection of the studies.

Of a total of 10,595 publications retrieved, 8 prospective studies [6,7,8,9,10,11,12,27] were identified that met the inclusion criteria (Figure 1).

However, one prospective study was excluded because it did not report useful data to perform dose–response analysis [27].

A total of seven studies were included in the meta-analysis of diabetes risk assessment [6,7,8,9,10,11,12] (Table 1).

Almost all studies were conducted in the US, one was conducted in Finland and the other one in China. The majority of the studies used a validated questionnaire as a proxy for K intake; one study provided risk estimates both by validated questionnaire and by 24 h urinary excretion of K separately; whilst only one utilized 24 h urine collection to assess K intake. All studies assessed diabetes at least by fasting blood glucose more than 126 mg/dL. In addition, some studies established criteria for diabetes as a non-fasting glucose of more than 200 mg/dL, and/or HbA1c more than 6.5%, and/or use of anti-diabetic medications. The mean dietary K intake varied from 1791 to 4120 mg/day. The length of follow-up ranged from 4.7 to 20 years, and the average age was from 25 to 72.9 years. The evaluation of the “risk of bias” indicated that all studies were substantially at low risk (Supplemental Table S2).

### 3.1. Dietary Potassium Intake and Risk of Diabetes

A total of seven studies were included for the analysis of K intake and risk of diabetes (overall, 31,873 participants and 4320 new diabetes cases) [6,7,8,9,10,11,12].

**Figure 2:**
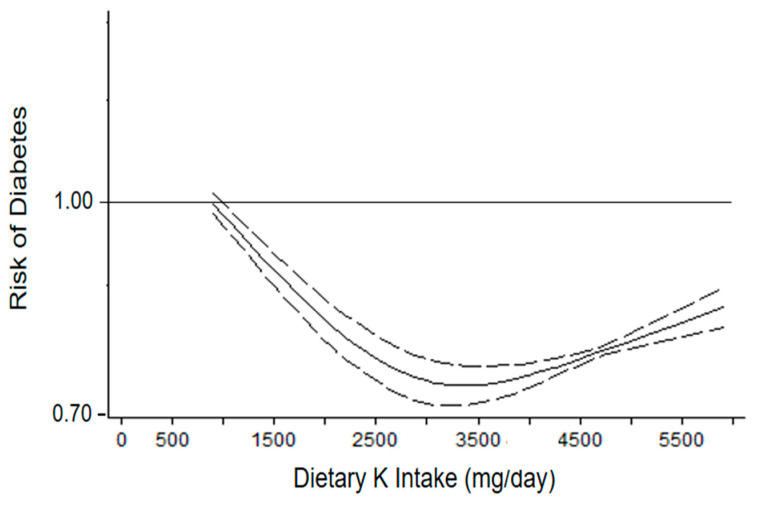
Dose–response association between potassium (K) intake and risk of diabetes (seven cohorts). Potassium intake was modelled with restricted cubic splines in a multivariate random-effects dose–response model (solid line). Dashed lines represent the 95% confidence intervals for the spline model.

The analysis of departure from linearity indicated a non-linear association between K intake and diabetes risk (*p* for non-linearity < 0.001) (Figure 2). In particular, the dose–response analysis showed a J-shape association, with a significant reduction in diabetes risk that started from K consumption of 1000 mg/day, and detected the lowest risk at 3300–3500 mg per day (HR = 0.80, 95%CI: 0.79 to 0.81) compared with lower consumption (lower than 1000 mg/day). By contrast, an opposite trend was found from 4000 mg per day but maintained the significant inverse relationship. Moreover, a significant heterogeneity was found (*p* < 0.01).

Additional analyses. Visual analysis of the funnel plot indicated asymmetry (Supplemental Figure S1), whereas Egger’s and Begg’s tests did not find significant evidence of publication bias (Egger’s test: *p* = 0.39; Begg’s test: *p* = 0.88). Additionally, the “trim and fill” method did not identify any possibly missing study.

Moreover, the univariate meta-regression analysis did not detect age (coeff. = 0.0031285; *p* = 0.2), gender (coeff. = 0.0079075; *p* = 0.4), length of follow-up (coeff. = −0.0050414; *p* = 0.5), total number of participants (coeff. = 2.19 × 10^−6^; *p* = 0.8), year of publication (coeff. = 0.0030065; *p* = 0.7), baseline K intake (coeff. = 2.21× 10^−6^; *p*= 0.9), baseline body weight (coeff. = 0.0143618; *p* = 0.6) and ethnicity (coeff. = −0.0008764; *p* = 0.4) as a significant source of heterogeneity (Supplemental Table S3).

Quality of body of evidence. According to the GRADE criteria, the evidence for the association between K intake and diabetes risk was of moderate quality. Despite the GRADE methodology defining observational evidence from cohort studies as low quality, there was an upgrade of the score due to the dose–response gradient.

#### 3.1.1. Urinary Potassium Excretion

Only two studies were included in the evaluation of the association between urinary K excretion and risk of diabetes (overall, 3001 participants and 228 new diabetes cases) [6,8] (Table 1).

**Figure 3:**
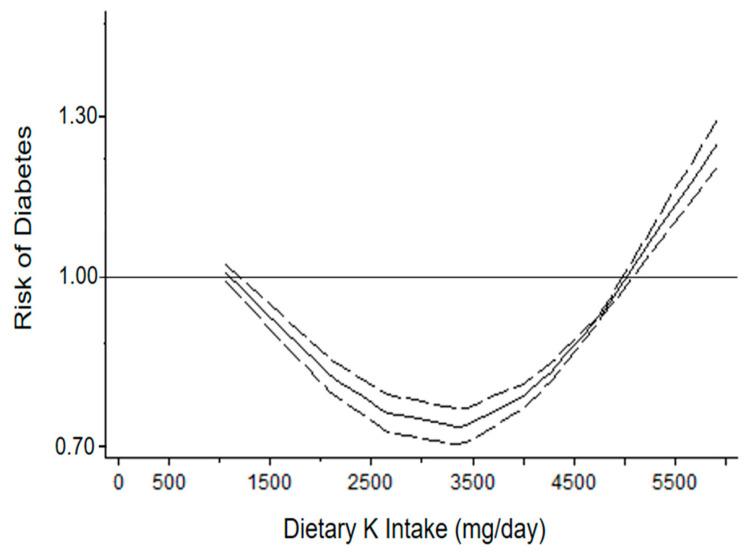
Dose–response association between dietary potassium (K) intake by urinary excretion and risk of diabetes (two cohorts). Urinary potassium excretion was modelled with restricted cubic splines in a multivariate random-effects dose–response model (solid line). Dashed lines represent the 95% confidence intervals for the spline model.

The dose–response analysis indicated a non-linear association (*p* for non-linearity < 0.001), in particular, a J-shape relationship between baseline urinary K excretion and the risk of diabetes was detected (Figure 3).

Urinary K excretion consumption from 40 up to 100 mmol per day (from 2030 to 5070 mg/day) was associated with a significantly lower risk of diabetes, with the lowest risk at 3350 mg/day (HR = 0.74, 95% CI: 0.73 to 0.76) compared with lower consumption (up to 1014 mg/day), while an opposite trend was found from 5880 mg per day) compared with lower intake. There was significant heterogeneity among studies (*p* < 0.01).

#### 3.1.2. Dietary Questionnaire

**Figure 4:**
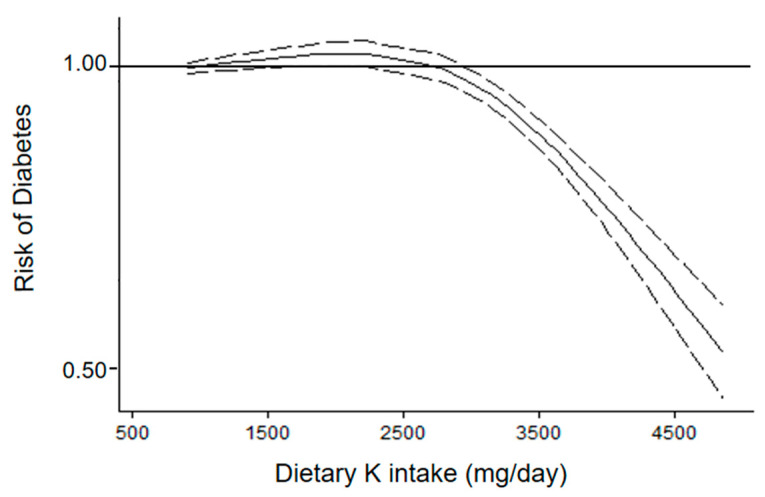
Dose–response association between dietary potassium (K) intake by questionnaire and risk of diabetes (six cohorts). Dietary potassium intake was modelled with restricted cubic splines in a multivariate random-effects dose–response model (solid line). Dashed lines represent the 95% confidence intervals for the spline model.

A total of six studies were available for the analysis of dietary K intake and risk of diabetes (overall, 33,626 participants and 4465 new diabetes cases) [7,8,9,10,11,12]. The analysis of departure from linearity [7,8,10,11,12] indicated a non-linear association between K intake and diabetes risk (*p* for non-linearity < 0.001) (Figure 4).

A consumption of K between 1500 and 2200 mg per day was significantly associated with a modest higher risk of diabetes compared with a consumption less than 1000 mg per day. By contrast, a consumption more than 2900 mg per day was significantly associated with lower risk of diabetes compared with a consumption less than 1000 mg/day (Figure 3). A significant heterogeneity was found (*p* < 0.01).

A further analysis including the study with risk expressed as OR [9], showed similar results (*p* for non-linearity < 0.001) (Supplemental Figure S2). Additionally, this analysis detected significant heterogeneity among studies (*p* < 0.01).

## 4. Discussion

The results of our meta-analysis indicate that dietary K intake is associated with risk of diabetes in general population. In particular, these prospective data suggest a J-shape association between K consumption and the rate of incident diabetes cases. The risk decreases from K intake of 1000 mg/day compared with lower consumption, with a 20% lower risk at 3300–3500 mg per day. These results were supported by the GRADE categorization that detected moderate quality. A not similar shape of association was detected after stratification by different K assessment, especially at high K intake, because of the possible misclassification of dietary intakes by questionnaire and the different highest thresholds of K intake between the two measurements. Nonetheless, there are concordant data on the inverse association at intermediate consumption, in particular, a significant beneficial effect starting from 2900 mg per day by questionnaires, and the greatest beneficial effect at ~3400-mg per day by urinary excretion.

The non-linear association between dietary K intake—in particular with urinary K excretion—and risk of diabetes may be due to phenomenon of hormesis [28]. Indeed, the beneficial effect detected at moderate K consumption may be explained by the favourable health effect of “low-moderate dose” bioactive substances. By contrast, at a high level of K consumption, the benefit may be offset by the unfavourable consequences of excess food consumption (e.g., increase in caloric intake).

Thirty years ago, an observational study explored the relationship between dietary K and diabetes risk [27]. This study showed an inverse association between K intake and the risk of diabetes. However, the study included only women and the models were adjusted for few covariates.

Following this study, other epidemiological evidence explored this topic with contrasting results and without evidence of a dose–response relationship. One of them found an inverse association between K intake and risk of diabetes by 24 h urinary K excretion in a cohort of young adults [8]; while low dietary K was associated with increased risk of diabetes only in African Americans when K intake was assessed by questionnaire [8]. Another American study found a significant inverse association between dietary K intake and diabetes risk in an unadjusted model, but not in models adjusted for main confounders [7]. By contrast, a European study that assessed the risk of diabetes by 24 h urinary excretion did not detect any association after 18 years of follow-up [6]. Likewise, other studies did not find any significant associations between K intake and diabetes risk by questionnaire [9,10,11,12].

Of note, our findings are at variance with a previous meta-analysis, in which K intake was not associated with diabetes risk both by questionnaire and urinary excretion [13]. The latter study had major limitations in the inclusion of a study that assessed urinary excretion of K by spot urine [12], in inclusion of a smaller number of studies for dietary K assessment by questionnaire, and in the lack of assessment of a potential dose–response relationship. Our relevant elements of novel information of the present meta-analysis include detailed dose–response analysis with detection of non-linear relationships, inclusion of new available data, stringent inclusion criteria, and assessment of the quality of the results by the GRADE categorization.

On the other hand, our results are substantially in agreement with a recent meta-analysis on K intake and blood pressure showing a non-linear relationship between K intake and changes in blood pressure [29].

Unfortunately, only two RCTs explored the glucose profile as a function of the K intake [15,16]. These two studies assessed K intake by a 24 h urine collection at baseline and at the end of intervention. Both included male and female participants with altered glucose metabolism and excess body weight. One study, including 27 middle-aged African American participants, found a significant reduction in fasting blood glucose after 12 weeks of K chloride (40 mEq K/day–1560 mg/day) compared with a placebo [16]. The other study, including 11 middle-aged participants, assessed the effect of K supplementation in K chloride (90 mEq K/day–3510 mg/day) or K citrate (90 mEq K/day–3510 mg/day) for 2 weeks [15]. Both interventions significantly increased insulin production compared with a placebo, but only K citrate improved markers of insulin resistance and sensitivity.

It is worthy of note that while there was a wide range of baseline average K intakes in the cohorts included (from 1791 to 4120 mg/day), only two cohorts [6,8] achieved the international recommendations [30]. However, these data seem not to affect the association between K intake and new-onset diabetes by meta-regression analysis.

### Strengths and Limitations

This study has several strengths: (a) stringent inclusion criteria; (b) a relatively large number of participants; (c) the evaluation of the shape and strength of the dose–response relationship; (d) the assessment of the overall quality of evidence using the GRADE assessment approach and (e) the comprehensive exploration of possible sources of heterogeneity.

Conversely, some limitations must be discussed. Their observational nature impairs conclusions about possible cause–effect relationships. Although several mechanisms are involved to contribute to a very tight balance between intra and extra-cellular K [31], experimental data suggest a relationship among K intake, serum K and insulin secretion and sensitivity by RAAS. For instance, K supplements had favourable impacts on insulin sensitivity [16,32] and improved glucose intolerance during low serum K [5,33].

Further limitations are given by the relatively small number of prospective studies and cohorts available, by the residual possibility of publication bias and by the difficulty to draw definitive conclusions with regard to the interaction among age, gender and race given the composition of the study cohorts available. This notwithstanding, the observational design and the process of meta-analysis, with the calculation of a pooled estimate of the effect and the dose–response analysis, and the meta-regression analysis are able to counterbalance some of these issues.

Another limitation may be that the majority of the studies included had the same prominent author. However, the analyses were performed on single different structured databases (i.e., ARIC, CARDIA, CHS, MESA and JHS), and various results were detected; therefore, a bias of the analyses can be ruled out.

Additionally, variable methods of the diagnosis of new-onset diabetes may be a limitation. Indeed, although in all studies included the diagnosis was assessed at least by fasting blood glucose more than 126 mg/dL, additional criteria were different.

The heterogeneity among studies is another limitation, nevertheless the main potential confounders explored (e.g., age, length of follow-up, body weight and gender) did not affect the relationship.

Finally, other factors, including the presence of genetic susceptibility, which may mediate associations between K and glucose metabolism, were not explored.

## 5. Conclusions

The results of our meta-analysis show that dietary K intake is associated with risk of diabetes in the general population with a J-shape relationship, in particular, with the apparent benefit against the development of diabetes at consumption comprised between 3000 and 5000 mg per day. These findings support the international recommendations on the increase in daily K consumption through regular fresh fruit and vegetable consumption in the general population to reduce cardiovascular risk [34,35], also in consideration of the low K intake at population level.

Given the importance of diabetes [17] and its implication in cardiovascular risk, and that international dietary guidelines do not express recommendations about K intake and glucose control and diabetes risk because of limited and conflicting data [14,36], the relationship found between dietary K and diabetes risk assumes considerable relevance. However, further properly powered RCTs of the effect of long-term moderate dietary K consumption or K supplementation are warranted to determine possible cause–effect relationships, to disentangle the effects of potential bias and those of other compounds of K rich foods [37,38] and to overcome the currently limited evidence with respect to the interactions among age, ethnicity and diseases. In particular, intervention studies with carefully controlled intake of K should evaluate the mechanisms of its effects on glucose metabolism, and in turn, the risk of diabetes to extend current knowledge in this field.

## Figures and Tables

**Table 1:** Characteristics of the studies included in the systematic review.

Prospective Studies										
First Author, Year (Ref)	Country	Population [Ethnicity]	Participants (n)/Cases (n)	Gender [M/F] (%)	Follow-Up (Year)	Age (Year) (Range)	BMI (kg/m^2^)	Dietary Assessment Tool	Dietary Potassium Intake (mg/day)	Outcome (Type of Assessment)
Hu, 2005 [6]	Finland	General population	1935/129	48/52	18.1	49.7 (35–64)	27	24 h urinary potassium excretion	4120 *	Fasting blood glucose > 126 mg/dL Non-fasting glucose > 200 mg/dL
Chatterjee, 2010 [7]	US	ARIC [African American 22%]	12,209/1475	44/56	9	54 (45–65)	27	Validated FFQ	2655	Fasting blood glucose ≥ 126 mg/dL Non-fasting glucose ≥ 200 mg/dL Clinician-diagnosed diabetes Use of antidiabetic medications
Chatterjee, 2012 [8]	US	CARDIA [African American 50%]	4754/373	45/55	20	25 (18–30)	24.5	Validated FFQ	3661	Fasting blood glucose ≥ 126 mg/dL Non fasting glucose ≥ 200 mg/dL HbA1c ≥ 6.5% Use of antidiabetic medication
		[African American 55%]	1066/99	43/57	15	30 (18–30)	26.9	24 h urinary potassium excretion	2788 *	
Chatterjee, 2015 [11]	US	CHS	4111/375	41/59	12.1	72.9 (≥65)	26.3	Validated FFQ	3191	Fasting blood glucose ≥ 126 mg/dL Non-fasting glucose ≥ 200 mg/dL Use of antidiabetic medications
Chatterjee, 2016 [10]	US	MESA [42% Caucasian/white, 24.8% African American, 20.5% Hispanic, 12.5% Asian]	5415/1281	46/54	8	61.8 (45–84)	28	Validated FFQ	2555	Fasting blood glucose ≥ 126 mg/dL Use of antidiabetic medications
Chatterjee, 2016 [12]	US	Jackson Heart Study [African American]	2157/398	37/63	8	52.4 (21–95)	31.1	Validated FFQ	2542	Fasting blood glucose ≥ 126 mg/dL HbA1c ≥ 6.5% Use of antidiabetic medications
Hao, 2020 [9]	China	CHNS	5867/611	46/54	4.7	48.1 (18–93)	**	3 consecutive 24 h recalls	1791	Fasting glucose levels ≥ 126 mg/dL HbA1c ≥ 6.5% Use of antidiabetic medications

ARIC: The Atherosclerosis Risk in Communities Study; CARDIA: The Coronary Artery Risk Development in Young Adults study; CHNS: China Health and Nutrition Survey; CHS: The Cardiovascular Health Study; MESA: Multi-Ethnic Study of Atherosclerosis; * conversion factor 1.3; ** 38% of obesity.

## Data Availability

Not applicable.

## Supplementary Materials

The following supporting information can be downloaded at: https://www.mdpi.com/article/10.3390/nu14224785/s1, Figure S1: Funnel plot of the association between dietary potassium intake and risk of diabetes; Figure S2: Dose–response association between dietary potassium (K) intake and risk of diabetes, also including Jackson cohort [9]. Dietary potassium intake was modelled with restricted cubic splines in a multivariate random-effects dose–response model (solid line). Dashed lines represent the 95% confidence intervals for the spline model; Table S1: The PRISMA Checklist; Table S2: Newcastle–Ottawa Quality Assessment Scale for cohort studies included in the meta-analysis; Table S3: Meta-regression analysis of the effect of dietary potassium intake on diabetes risk.
